# Comparative Efficacy of Azithromycin and Clarithromycin in the Management of Helicobacter pylori Infection

**DOI:** 10.7759/cureus.72033

**Published:** 2024-10-21

**Authors:** Sami H Alhijazien, Shadi Daoud, Marwan T Alzayadi, Majd R Al Sarhan, Moayad K Aldomi, Talal A Al Shawabkeh, Mohammad M Alsmadi

**Affiliations:** 1 Gastroenterology and Hepatology, Jordanian Royal Medical Services, Amman, JOR; 2 Rheumatology, Jordanian Royal Medical Services, Amman, JOR; 3 Internal Medicine, Jordanian Royal Medical Services, Amman, JOR

**Keywords:** azithromycin, clarithromycin, h. pylori eradication, macrolide-based regimens, peptic ulcer diseases

## Abstract

Aim: In this study, the main goal was to find a statistically significant difference between eradication treatment plans that used azithromycin and clarithromycin in triple and quadruple eradication plans.

Methods: This retrospective, single-center, observational, non-funded Helicobacter pylori management study examined patients who visited our institutional gastroenterology clinic at the King Hussein Medical Centre in the Royal Medical Services, Amman, Jordan, from January 2023 to May 2024. The most common treatment (TRT) courses are 7 or 10-14 days, so this study divided the therapy length into two categories. We also divided proton pump inhibitors (PPIs) into low-standard dose intensity (omeprazole 20-40 mg/day) and high-dose intensity (80 mg/day). Patients received six TRT regimens. The study aimed to eradicate H. pylori with a negative stool antigen test after six weeks post-TRT and two weeks without PPIs. Clarithromycin and azithromycin were reference macrolides for TRT. Azithromycin-based TRT covered IV-VI, while clarithromycin-based TRT covered I-III. The chi-square test was used to quantify TRT I-II proportional differences in patient variables. Association coefficients, odd ratios for TRT success, prediction variability range, and H. pylori eradication sensitivity indices based on macrolides-based TRT I-II, TRT length categories, PPI intensity doses, and patients' adherence rating scale were extracted using multiple logistic regression.

Results: Our gastroenterology unit tested 1076 patients. About 49.3% (530 patients) were female and 50.7% (546 patients) were male. The majority of patients, 78.2% (841), were under 60, while 21.8% (235) were 60 or older. Patients were given six eradication regimens (Regimens I-VI) with macrolides (clarithromycin or azithromycin). Most TRT durations were 7-14 days. We used multiple logistic regression. We considered patient adherence rates as confounding factors. Using azithromycin instead of clarithromycin has a statistically significant impact (1.780 (95% CI; 1.378-2.299).

Conclusion: Azithromycin can be a reasonable substitute for clarithromycin in triple or quadruple therapy eradication regimens for H. pylori infection.

## Introduction

Helicobacter pylori infection is a common bacterial infection affecting 30-50% of the global population, with Asian countries having the highest prevalence and low socioeconomic status. H. pylori is a Gram-negative bacterium found in the mucus layer of the human stomach, with the highest prevalence in developing countries. It is the leading cause of gastritis and peptic ulcer disease and a risk factor for gastric cancer [1]. The bacterium is acquired through person-to-person contact in early childhood and is lifelong unless treated. Some disorders outside of the stomach are more likely to happen if you have H. pylori, and 10-15% of people who have it get serious problems in their stomach and duodenum, like peptic ulcer disease or gastric adenocarcinoma [2]. The invasive and non-specific nature of gastric biopsies for detecting H. pylori infection, as well as basal signs or symptoms, often allows for a late diagnosis of H. pylori-associated diseases, further complicating their treatment [3]. H. pylori epidemiology and transmission are critical for formulating public health strategies and interventions. The most severe manifestations of H. pylori-related gastric histological abnormality are the occurrence of premalignant conditions, atrophy, and intestinal metaplasia [4]. Gastric adenocarcinoma is the fifth most prevalent cancer worldwide [5].

Treatment strategies include anti-helicobacter drugs like proton pump inhibitors (PPIs), clarithromycin, amoxicillin, metronidazole, and tetracycline. However, increasing drug resistance has led to a decline in eradication rates [6]. Current guidelines recommend amoxicillin-containing triple or quadruple therapy for 7-14 days as the first-line treatment, and bismuth quadruple treatments for second-line rescue. Common regimens include amoxicillin, clarithromycin, metronidazole, levofloxacin, and tetracycline-based triple or quadruple treatment eradication regimens [7]. These treatments can reach an eradication rate of up to 90%. However, over the past two decades, the eradication rate of these first-recommended lines has been declining, leading to a more diverse and complex treatment landscape that requires new effective treatment regimens and future directions to address the multifactorial pathogenicity of H. pylori. Investing in research and personalized treatment protocols is critical for achieving reliable treatment outcomes [8,9].

Clarithromycin and azithromycin are macrolide antibiotics, with the latter providing wider coverage. Both antibiotics are acid-stable, contributing to their pharmacokinetics. Clarithromycin is one of the first lines of eradication treatment for H. pylori infection, and its clinical efficacy is generally significant [10]. The recommended dose for H. pylori eradication therapy is 500-1000 mg per dose daily, with the usual on-label recommended dose being 500 mg twice a day or an off-label dose of 1000 mg once daily with omeprazole and amoxicillin [11]. International guidelines use azithromycin, a macrolide antibiotic, as a second-line therapy after clarithromycin-based first-line therapy fails. It has high efficacy, safety, tolerability, and patient compliance. However, due to limited evidence, not all countries have approved it [12].

In this study, our primary goal was to identify a statistically significant difference between adopting an azithromycin-based eradication regimen and using clarithromycin-based eradication regimens in both triple and quadrable eradication regimens. We also examined the numerous variations in clinical practice, including the length of the regimens (7, 10, or 14 days), the strength of the PPIs (low, standard, or high), and the varying levels of medication adherence.

## Materials and methods

From January 2023 to May 2024, this retrospective, single-center, observational, non-funded study on H. pylori management focused on patients who visited our institutional gastroenterology clinic at the King Hussein Medical Centre in the Royal Medical Services, Amman, Jordan. This study was initially approved by our Jordanian Institutional Review Board (JIRB) on 30 July 2024 under registration number 19_11/2024 and finalized on 21 August 2024. It follows the 1975 Declaration of Helsinki.

The study included eligible symptomatic H. pylori patients and used valid confirmatory testing. In our institution, H. pylori is diagnosed by H. pylori stool antigen (HPSA) positivity. Endoscopic procedures weren't performed according to hospital protocol unless there were alert risk factors like unexplained weight loss, coffee ground vomitus, persistent dyspepsia despite PPIs, iron deficiency anemia, dysphagia, early and prolonged satiety, and a positive family history of upper gastrointestinal carcinoma.

Importantly, this study collected patients' gender, age, pre-treatment symptomatic duration, stool test positivity for H. pylori antigen, endoscopic procedure, history of penicillin allergy, H. pylori eradication regimen, PPI intensity dose, compliance, duration, socioeconomic status, eating restaurant food, meat consumption, and smoking status. Our institutional EMR system stored these retrievable data from patient-gastroenterologist interview notes. Patients with 20% missing data and discordance were excluded. Additionally, patients under 15 years old were excluded from the study. Taking 80% of prescribed drugs was considered adequate treatment compliance.

Based on our institution's most commonly prescribed recommended and investigational treatments, the attended patients were divided into six therapeutic regimens (Regimens I-VI). Regimens I-III were Standard Treatments (Stratum I) and Regimens IV-VI were Investigational Treatments (Stratum II). Triple therapy with PPI, clarithromycin, and metronidazole (Regimen I); Triple therapy with PPI, clarithromycin, and amoxicillin (Regimen II); Quadruple concomitant therapy with PPI, clarithromycin, amoxicillin, and metronidazole (Regimen III); Triple therapy with PPI, azithromycin, and amoxicillin (Regimen IV); Triple therapy with PPI, azithromycin, and metronidazole (Regimen V); and Triple therapy with PPI, azithromycin, and higher-amoxicillin dose (Regimen VI). Regimens I-VI used 1 gram BID amoxicillin (except Regimen VI, which used 2 g BID) for 7-14 days, 500 mg BID clarithromycin for 7-14 days, 500 mg BID metronidazole for 7-14 days, and 250 mg BID azithromycin for seven days. Omeprazole or esomeprazole was given at 20-80 mg per day in this study. Although 20 mg of omeprazole per day was considered low, 40 mg and 80 mg were considered standard and high, respectively.

This study divided the therapy length into binary levels based on the most common durations: seven days (shorter TRT course) or 10-14 days longer TRT course). We also divided PPIs into low-standard dose intensity (omeprazole 20-40 mg/day) and high-dose intensity (omeprazole 80 mg/day). The 10-item self-reporting multidimensional Medication Adherence Rating Scale (MARS) measures medication adherence behavior (1-4), attitude towards medication (5-8), and negative side effects and attitudes to psychotropic medication (9-10). Questions 1-6 and 9-10 code no responses as 1, while questions 7 and 8 code yes responses as 1. A higher MARS score indicates better medication adherence [13].

This study's primary goal was H. pylori eradication success, defined as a negative HPSA test after at least six weeks post-TRT regimens and two weeks without PPIs. This study examined TRT regimens with clarithromycin or azithromycin as the reference macrolides. Clarithromycin-based TRT regimens covered TRT I-III, while azithromycin-based regimens covered TRT IV-VI.

The chi-square test was used to analyze proportional differences in patient variables across TRT I-II. The OR and 95% confidence intervals were compared to a reference category. Multiple logistic regression analysis was used to extract association coefficients, odd ratios for TRT success, prediction variability range, and sensitivity indices for H. pylori eradication based on macrolide-based TRT I-II, TRT length categories, PPI intensity doses, and patients' MARS-10 score. Also plotted were MARS-10 regression associations against the probability for H. pylori post-TRT eradication across clarithromycin-based TRT regimens versus Azithromycin-based TRT regimens, taking into account four possible TRT lengths and PPI intensities: longer TRT lengths and higher PPI intensities, longer TRT lengths and low-standard PPI intensities, and shorter TRT lengths and higher PPI intensities.

Microsoft Excel 20 (Microsoft Corporation, Redmond, USA) was used to collect, summarize, and filter data. IBM SPSS Statistics for Windows, Version 25 (Released 2017; IBM Corp., Armonk, New York, United States) was used to analyze data. This study used 0.05 as the statistical significance threshold.

## Results

A total of 1076 patients were tested at our gastroenterology unit. Of these, approximately 49.3% (530 patients) were female and 50.7% (546 patients) were male. The majority of patients, 78.2% (841 patients), were under the age of 60, while only 21.8% (235 patients) were 60 years or older. Among the tested cohort, only 48.7% (524 patients) tested positive for H. pylori before treatment. Approximately 24.9% (268 patients) underwent an endoscopic procedure (EDG) to confirm H. pylori infection, as they exhibited alarming symptoms. Of these patients, 54.7% (157 patients) were diagnosed with H. pylori-related ulcer based on the biopsy results.

Approximately 49.35% (531 patients) received clarithromycin 500 mg twice daily for 7-14 days as the chosen macrolide-based eradication regimen (Regimen I), while approximately 50.65% (545 patients) were given azithromycin 250 mg once daily for seven days as the chosen macrolide-based eradication regimen (Regimen II). Approximately 57.9% (623 patients) successfully achieved the post-treatment goal of eradicating H. pylori infection, as indicated by a negative HPSA test result. This was observed after six weeks of completing the treatment regimen and at least two weeks after discontinuing PPIs. Conversely, a total of 42.1% (453 patients) did not meet the post-TRT target of achievement, as indicated by the HPSA positivity test. The success rates of post-macrolide-based TRT were significantly observed in approximately 51.4% (273 patients) and 64.2% (350 patients) in the clarithromycin and azithromycin-based TRT regimens, respectively. The odds ratio for these macrolide-based TRT regimens was reported as 1.696 (95% CI; 1.329-2.165).

A total of six eradication regimens (Regimens I-VI) were administered to the treated patients, as follows: Out of the total number of patients, 186 (35%) were treated with a combination of a PPI, clarithromycin, and metronidazole in Regimen I. In Regimen II, 184 patients (34.7%) received triple therapy with PPI, clarithromycin, and amoxicillin. Regimen III involved quadruple concomitant therapy with PPI, clarithromycin, amoxicillin, and metronidazole, and it was administered to 161 patients (30.3%). In Regimen IV, 173 patients (31.7%) were treated with a triple therapy consisting of PPI, azithromycin, and amoxicillin. Similarly, Regimen V involved a triple therapy with PPI, azithromycin, and metronidazole, and it was given to 193 patients (35.4%). Lastly, in Regimen VI, 179 patients (32.8%) received a triple therapy with PPI, a higher dose of amoxicillin, and azithromycin.

The majority of treatment durations fell into the longer TRT duration category (7-14 days) as opposed to the shorter TRT duration (7 days) [711 (66.1%) vs 365 (33.9%), respectively]. The TRT eradication regimens (Regimen I-VI) used low-standard PPI intensities (equivalent to 20-40 mg of either omeprazole or esomeprazole per day) in 69.6% of cases, compared to higher PPI intensities (equivalent to 80 mg of either omeprazole or esomeprazole) in 30.4% of cases (749 vs 327, respectively). The distribution rates of the patients studied were statistically insignificant across the two macrolide-based treatment regimens being compared: clarithromycin-based regimen (Regimen I) and azithromycin-based regimen (Regimen II), except for the post-treatment success rates. Both omeprazole and esomeprazole, which are both PPIs, were used in a similar manner in the patients we observed and studied. The distribution rate comparisons for the patients' tested variables across both treatment regimens are presented in Tables 1, 2.

**Table 1:** Comparisons of patients' demographic and diagnostic variables across macrolide-based eradication regimens A chi-square test was conducted in this study to express the distribution rates, odd ratios, and level of significance for the patients' above-tested variables. TRT: Treatment; F: Female; M: Male; OR: Odds ratio; Yrs: Years; HPSA: H. pylori stool antigen; EDG: Esophagogastroduodenoscopy; NA: Statistically not applicable.

	Clarithromycin-based TRT Regimens (Regimen I)	Azithromycin-based TRT Regimens (Regimen II)	Total	OR	p-Value
	(531, 49.35%)	(545, 50.65%)	1076		
Gender					
F	258 (48.6%)	272 (49.9%)	530 (49.3%)	0.949 (95% CI; 0.747-1.205)	0.665
M	273 (51.4%)	273 ((50.1%)	546 (50.7%)		
Age (Yrs)					
<60	410 (77.2%)	431 (79.1%)	841 (78.2%)	0.896 (95% CI; 0.671-1.197)	0.458
≥60	121 (22.8%)	114 (20.9%)	235 (21.8%)		
Pre_TRT_HPSA_Positivity					
Negative	274 (51.6%)	278 (51.0%)	552 (51.3%)	1.024 (95% CI; 0.806-1.301)	0.846
Positive	257 (48.4%)	267 (49.0%)	524 (48.7%)		
Alarming signs					
0	204 (38.4%)	210 (38.5%)	414 (38.5%)	NA	0.467
1	70 (13.2%)	54 (9.9%)	124 (11.5%)		
2	70 (13.2%)	72 (13.2%)	142 (13.2%)		
3	55 (10.4%)	73 (13.4%)	128 (11.9%)		
4	61 (11.5%)	62 (11.4%)	123 (11.4%)		
5	71 (13.4%)	74 (13.6%)	145 (13.5%)		
EGD					
No	399 (75.1%)	409 (75.0%)	808 (75.1%)	1.005 (95% CI; 0.762-1.325)	0.971
Yes	132 (24.9%)	136 (25.0%)	268 (24.9%)		
Pre_TRT_Biopsy_Positivity					
Negative	68 (48.9%)	62 (41.9%)	130 (45.3%)	1.328 (95% CI; 0.834-2.117)	0.232
Positive	71 (51.1%)	86 (58.1%)	157 (54.7%)		

**Table 2:** Comparison of patients' TRT-related variables for eradication treatment across the two macrolide-based regimens A chi-square test was conducted in this study to express the distribution rates, odd ratios, and p-values for the patients' treatment (TRT)-related investigated variables. Triple therapy with PPI, clarithromycin, and metronidazole (Regimen I); Triple therapy with PPI, clarithromycin, and amoxicillin (Regimen II); Quadruple concomitant therapy with PPI, clarithromycin, amoxicillin, and metronidazole (Regimen III); Triple therapy with PPI, azithromycin, and amoxicillin (Regimen IV); Triple therapy with PPI, azithromycin, and metronidazole (Regimen V); and Triple therapy with PPI, azithromycin, and higher-amoxicillin dose (Regimen VI). Regimens I-VI used 1 gram BID amoxicillin (except Regimen VI, which used 2 g BID) for 7-14 days, 500 mg BID clarithromycin for 7-14 days, 500 mg BID metronidazole for 7-14 days, and 250 mg BID azithromycin for seven days. Omeprazole or esomeprazole was given at 20-80 mg per day in this study. Although 20 mg of omeprazole per day was considered low, 40 mg and 80 mg were considered standard and high, respectively. TRT: Treatment; OR: Odds ratio; PPIs: Proton pump inhibitors; NA: Statistically not applicable.

	Clarithromycin-based TRT Regimens (Regimen I)	Azithromycin-based TRT Regimens (Regimen II)	Total	OR	p-Value
	(531, 49.35%)	(545, 50.65%)	1076		
TRT Regimens					
I	186 (35.0%)	0 (0.0%)	186 (17.3%)	NA	0.000
II	184 (34.7%)	0 (0.0%)	184 (17.1%)		
III	161 (30.3%)	0 (0.0%)	161 (15.0%)		
IV	0 (0.0%)	173 (31.7%)	173 (16.1%)		
V	0 (0.0%)	193 (35.4%)	193 (17.9%)		
VI	0 (0.0%)	179 (32.8%)	179 (16.6%)		
TRT duration (days)					
7	186 (35.0%)	179 (32.8%)	365 (33.9%)	NA	0.668
10	175 (33.0%)	179 (32.8%)	354 (32.9%)		
14	170 (32.0%)	187 (34.3%)	357 (33.2%)		
TRT duration (days)					
7	186 (35.0%)	179 (32.8%)	365 (33.9%)	1.102 (95% CI; 0.856-1.419)	0.449
10-14	345 (65.0%)	366 (67.2%)	711 (66.1%)		
PPI Intensity					
Low	183 (34.5%)	189 (34.7%)	372 (34.6%)	NA	0.857
Standard	190 (35.8%)	187 (34.3%)	377 (35.0%)		
High	158 (29.8%)	169 (31.0%)	327 (30.4%)		
PPI Intensity					
Low-Standard	373 (70.2%)	376 (69.0%)	749 (69.6%)	1.061 (95% CI; 0.818-1.376)	0.655
High	158 (29.8%)	169 (31.0%)	327 (30.4%)		
PPI Type					
Omeprazole	282 (53.1%)	262 (48.1%)	544 (50.6%)	1.223 (95% CI; 0.963-1.554)	0.099
Esomeprazole	249 (46.9%)	283 (51.9%)	532 (49.4%)		
Post-TRT Success					
Failure	258 (48.6%)	195 (35.8%)	453 (42.1%)	1.696 (95% CI; 1.329-2.165)	0.000
Success	273 (51.4%)	350 (64.2%)	623 (57.9%)		

In order to examine the relationships between the selected macrolide-based treatment regimen (clarithromycin vs azithromycin), the duration of treatment (shorter vs longer), the intensity of PPIs (slow-standard vs high), and the likelihood of achieving the desired outcome of post-treatment eradication success, we conducted a multiple logistic regression analysis. We also took into account the patients' adherence rates as potential confounding factors. Our findings indicate that adopting azithromycin instead of clarithromycin has a statistically significant impact, with a value of 1.780 (95% CI; 1.378-2.299). This impact is positively associated with the likelihood of achieving eradication, with a magnitude of 0.576±0.131, while controlling for other potential confounding factors. However, the influence of other tested potential factors was in favor of achieving the goal of eradicating the state after the end of treatment by at least six weeks and discontinuing PPIs for at least two weeks. The magnitude coefficients were highest for adopting higher PPI intensity compared to low-standard intensity [0.718±0.146], followed by adopting a TRT duration of 10-14 days compared to a shorter duration of seven days [0.543±0.137], and lastly by the medication adherence rating scale for each point [0.298±0.040].

The multiple regression model was constructed as follows: the probability of post-TRT success= [e^(-2.727+0.576×M+0.543×D+0.718×I+0.298×A)]/[(1+e^(-2.727+0.576×M+0.543×D+0.718×I+0.298×A)]. In this equation, M represents the macrolide adopted (0 for clarithromycin and 1 for azithromycin), D represents the TRT duration (0 for 7 days and 1 for 10-14 days), I represents the PPI intensity (0 for low-standard dose and 1 for high dose), and A represents the MARS 10 points.

This study uncovered that the predicted range of variability for the likelihood of eradicating H. pylori was found to be between 9.85% and 13.2%, depending on the methods of Cox & Snell and Nagelkerke R squared that were utilized [14].

**Figure 1:**
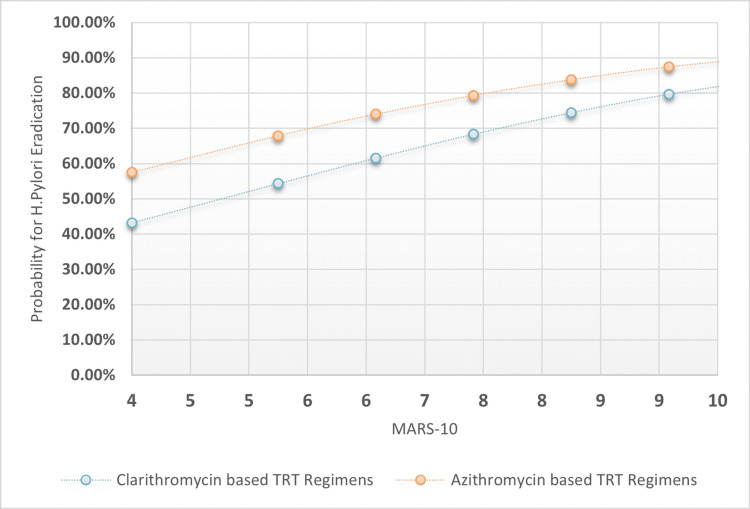
MARS-10 regressional associations against the probability for H. pylori post-TRT eradication across clarithromycin-based TRT regimens versus azithromycin-based TRT regimens when the tested patients adopted high PPI intensity rather than low-standard intensities and the TRT duration at least 10 days MARS 10: Medication adherence rating scale from 0-10 points; H: Helicobacter; TRT: Treatment; PPI: Proton pump inhibitor

**Figure 2:**
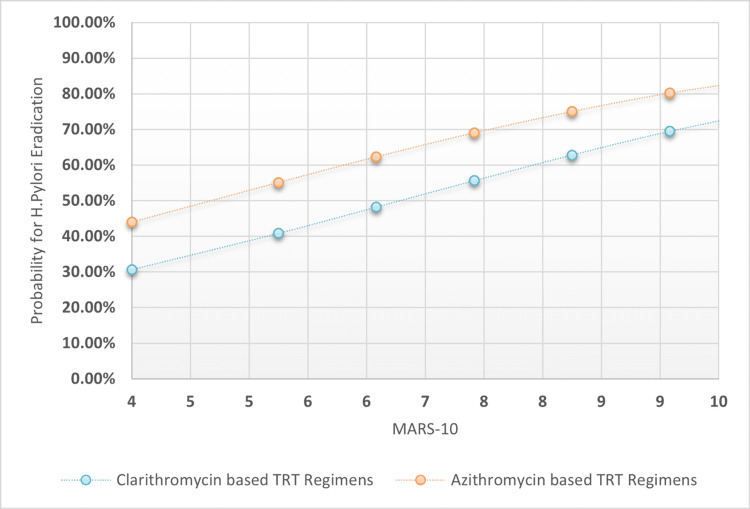
MARS-10 regressional associations against the probability for H. pylori post-TRT eradication across clarithromycin-based TRT regimens versus azithromycin-based TRT regimens when the tested patients adopted low-standard PPI intensities rather than high intensity and the TRT duration was seven days MARS 10: Medication adherence rating scale 10 points; TRT: Treatment; H. pylori: Helicobacter pylori; PPI: Proton pump inhibitor

**Figure 3:**
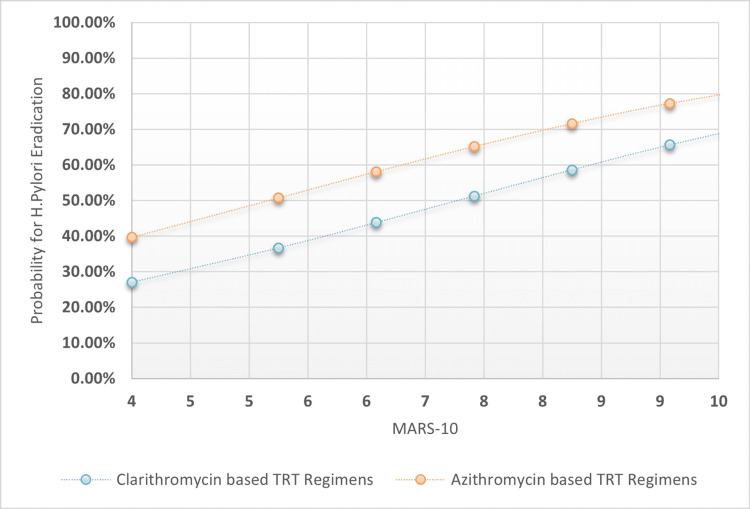
MARS-10 regressional associations against the probability for H. pylori post-TRT eradication across clarithromycin-based TRT regimens versus azithromycin-based TRT regimens when the tested patients adopted PPI low-standard intensities rather than high intensity and the TRT duration was at least 10 days MARS 10: Medication adherence rating scale from 0-10 points; H: Helicobacter; TRT: Treatment; PPI: Proton pump inhibitor

**Figure 4:**
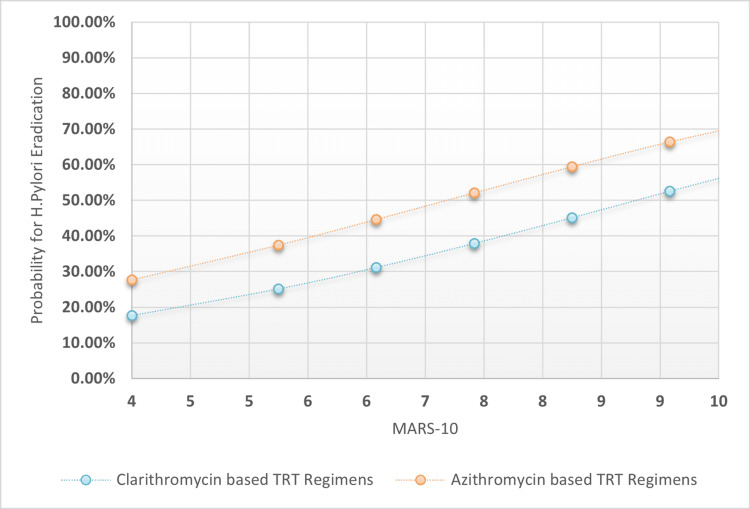
MARS-10 regressional associations against the probability for H. pylori post-TRT eradication across clarithromycin-based TRT regimens versus azithromycin-based TRT regimens when the tested patients adopted low-standard PPI intensities rather than high intensity and the TRT duration was seven days MARS 10: Medication adherence rating scale from 0-10 points; H: Helicobacter; TRT: Treatment; PPI: Proton pump inhibitor

The overall specificity was found to be 45.9%, with higher sensitivity rates of 77.2% and a moderately acceptable accuracy index of 64.0%. The positive predictive value was 50.94% and the negative predictive value was 68.27%. The statistical outcomes of the multiple logistic regression analysis are presented in Table 3. Additionally, Figures 1-4 depict four distinct regression plots that demonstrate the correlation between patients' MARS and the likelihood of achieving post-TRT eradication. These plots compare the effectiveness of two macrolide-based TRT regimens. In these various logistic regression analyses, we compared the probabilities of adherence patterns in patients receiving a clarithromycin-based TRT regimen versus an azithromycin-based regimen, as measured by the Medication Adherence Report Scale (MARS-10). The four possibilities considered the statuses of the dichotomized variables, which were TRT durations in days (0 for shorter duration and 1 for longer duration) and PPI intensity (0 for low intensity and 1 for high intensity).

**Table 3:** Statistical outcomes of the performed multiple logistic regression B: Regressional coefficient; SE: Standard error; EXP (B): Exponential of the regressional coefficient or impactful effect or estimated size; CI: Confidence interval; ABs: Antibiotics (clarithromycin or azithromycin in this study); TRT: Treatment; MARS10: Medication adherence rating scale from 0 to 10 points; PPI: Proton pump inhibitor

	B±S.E.	Sig.	Exp(B)	95% C.I.for EXP(B)	
ABs	0.576±0.131	0.000	1.780	(95% CI; 1.378-2.299)	
TRT_Duration	0.543±0.137	0.000	1.721	(95% CI; 1.315-2.252)	
PPIs_Intensity	0.718±0.146	0.000	2.051	(95% CI; 1.540-2.731)	
MARS10	0.298±0.040	0.000	1.346	(95% CI; 1.245-1.456)	
Constant	-2.727±0.337	0.000	0.065		

## Discussion

Triple therapy has become less popular, leading to the use of quadruple regimens for treating H. pylori infection. Researchers have found that bismuth-containing quadruple therapy and concomitant therapy have a higher eradication rate compared to standard therapy. Some experts advocate for 14-day fluoroquinolone-containing triple therapy as a first-line therapy for some patients with low to moderate clarithromycin resistance. A recent network meta-analysis found that 10-day, 14-day, and 7-day BQT have similar efficacies, but 10-day and 14-day CQT regimens show better results compared to 7-day regimens. Salvage therapy also recommends quadruple therapy, but the difference in treatment efficacy between salvage therapy and triple therapy is still unclear [15-17].

Not enough research has been done to fully compare how well azithromycin-based treatments work to standard triple or quadruple therapies, which usually use clarithromycin as the macrolide in their plans to get rid of H. pylori. This study investigated the differences in the likelihood of eliminating H. pylori when standard eradication protocols use azithromycin as the macrolide instead of clarithromycin. The study looked at what happened when the TRT was longer or shorter and when the PPI intensity was higher or lower. It also looked at other important possible confounding factors that are important for figuring out how likely it is that an H. pylori infection will be gone after taking a PPI and two antibiotics.

Various factors, including age, mental health, and medication availability, influence medication adherence to H. pylori eradication. Non-adherent patients are typically young male non-smokers with multiple drug regimens, depression, or gastrointestinal morbidity. We should determine strategies to improve medication adherence based on the patient's adherence status. Several large-scale RCTs have observed a favorable cure rate for non-bismuth quadruple regimens, such as concomitant therapy, hybrid therapy, and bismuth-based quadruple regimens for first-line H. pylori therapy. Some consensus recommends non-bismuth quadruple therapy as the first-line eradication therapy in countries with alarmingly high antibiotic resistance. Key studies and meta-analyses in the field of H. pylori eradication therapy are of significant importance for understanding the process and challenges that researchers in the area are facing today [18-20].

Tauqeer et al. compared triple therapies for H. pylori eradication in active duodenal ulcer patients. Researchers investigated 60 adults over 18 with active duodenal ulcers and H. pylori infection. One group received azithromycin, amoxicillin, and omeprazole, while the other received metronidazole. OAA triple therapy was more effective than OAM triple therapy, with age, gender, and symptom duration playing major roles [21].

In a 46,160-patient Korean study by et al., H. pylori clarithromycin resistance was 16.2%. The study independently linked resistance to female sex, age >50, use of macrolide antibiotics, and respiratory comorbidity. The study discovered that macrolide antibiotics and recent respiratory disease may increase H. pylori resistance [22].

Roberts et al. looked into how well adding nitazoxanide to a triple therapy based on clarithromycin worked for H. pylori [23]. Nitazoxanide, amoxicillin, clarithromycin, and esomeprazole comprised the 14-day NACE regimen. Eradication was safe and well-tolerated in 93.7% of 111 patients. Metwally et al. studied H. pylori antibiotic resistance in 134 adult patients with upper gastrointestinal complaints in Egypt. H. pylori was 10% resistant to metronidazole and amoxicillin, 15% to erythromycin, azithromycin, and clarithromycin, and 20% to levofloxacin [24].

The study revealed high resistance rates for amoxicillin/clarithromycin and amoxicillin/metronidazole, indicating a prioritization of quinolones. Bujanda et al. analyzed the time trend of antibiotic resistance in Europe using the European Registry on Helicobacter pylori Management (Hp-EuReg). From 2013-2016 to 2017-2020, the number of positive cultures dropped 35%. Most resistances were to metronidazole (30%), clarithromycin (25%), and levofloxacin (20%), with tetracycline and amoxicillin below 1%. Between 2013-2016 and 2017-2020, metronidazole resistance decreased [25].

This study has several limitations, including its retrospective design, single-center observational nature, relatively small sample size. To establish causality and address the probability of eradicating H. pylori when comparing azithromycin to clarithromycin, a multicenter randomized controlled double-blind study is needed. This study should also consider other potential factors such as the intensity and duration of PPI regimens, as well as patient adherence rates.

## Conclusions

In this study, we found that using a daily dose of 250 mg of azithromycin for seven days may be more effective in eradicating H. pylori compared to the standard clarithromycin-based therapy. This efficacy is further enhanced when the duration of treatment and the intensity of PPIs are prolonged and increased, respectively.
